# Significant Benefits of Environmentally Friendly Hydrosols from *Tropaeolum majus* L. Seeds with Multiple Biological Activities

**DOI:** 10.3390/plants12223897

**Published:** 2023-11-18

**Authors:** Ivana Vrca, Blaž Jug, Željana Fredotović, Elma Vuko, Valentina Brkan, Loriana Šestić, Lea Juretić, Valerija Dunkić, Marija Nazlić, Dina Ramić, Sonja Smole Možina, Dario Kremer

**Affiliations:** 1 Faculty of Science, University of Split, Ruđera Boškovića 33, 21000 Split, Croatia; zfredotov@pmfst.hr (Ž.F.); elma@pmfst.hr (E.V.); vbrkan@pmfst.hr (V.B.); lsestic@pmfst.hr (L.Š.); dunkic@pmfst.hr (V.D.); mnazlic@pmfst.hr (M.N.); 2 Biotechnical Faculty, University of Ljubljana, Jamnikarjeva ulica 101, 1000 Ljubljana, Slovenia; blaz.jug@bf.uni-lj.si (B.J.); dina.ramic12@gmail.com (D.R.); sonja.smole-mozina@bf.uni-lj.si (S.S.M.); 3 Faculty of Medicine, University of Rijeka, Braće Branchetta 20, 51000 Rijeka, Croatia; lea.juretic@medri.uniri.hr; 4 Faculty of Pharmacy and Biochemistry, University of Zagreb, Ante Kovačića 1, 10000 Zagreb, Croatia; dkremer@pharma.hr

**Keywords:** *Tropaeolum majus* L., microwave-assisted extraction, hydrosols, benzyl isothiocyanate, cytotoxic activity, scratch assay, antibacterial activity, antiadhesive activity, antiphytoviral activity

## Abstract

*Tropaeolum majus* L. is a traditional medicinal plant with a wide range of biological activities due to the degradation products of the glucosinolate glucotropaeolin. Therefore, the goals of this study were to identify volatiles using gas chromatography–mass spectrometry analysis (GC-MS) of the hydrosols (HYs) isolated using microwave-assisted extraction (MAE) and microwave hydrodiffusion and gravity (MHG). Cytotoxic activity was tested against a cervical cancer cell line (HeLa), human colon cancer cell line (HCT116), human osteosarcoma cell line (U2OS), and healthy cell line (RPE1). The effect on wound healing was investigated using human keratinocyte cells (HaCaT), while the antibacterial activity of the HYs was tested against growth and adhesion to a polystyrene surface of *Staphylococcus aureus* and *Escherichia coli*. Antiphytoviral activity against tobacco mosaic virus (TMV) was determined. The GC-MS analysis showed that the two main compounds in the HYs of *T. majus* are benzyl isothiocyanate (BITC) and benzyl cyanide (BCN) using the MAE (62.29% BITC and 15.02% BCN) and MHG (17.89% BITC and 65.33% BCN) extraction techniques. The HYs obtained using MAE showed better cytotoxic activity against the tested cancer cell lines (IC_50_ value of 472.61–637.07 µg/mL) compared to the HYs obtained using MHG (IC_50_ value of 719.01–1307.03 μg/mL). Both concentrations (5 and 20 µg/mL) of *T. majus* HYs using MAE showed a mild but statistically non-significant effect in promoting gap closure compared with untreated cells, whereas the *T. majus* HY isolated using MHG at a concentration of 15 µg/mL showed a statistically significant negative effect on wound healing. The test showed that the MIC concentration was above 0.5 mg/mL for the HY isolated using MAE, and 2 mg/mL for the HY isolated using MHG. The HY isolated using MHG reduced the adhesion of *E. coli* at a concentration of 2 mg/mL, while it also reduced the adhesion of *S. aureus* at a concentration of 1 mg/mL. Both hydrosols showed excellent antiphytoviral activity against TMV, achieving100% inhibition of local lesions on the leaves of infected plants, which is the first time such a result was obtained with a hydrosol treatment. Due to the antiphytoviral activity results, hydrosols of *T. majus* have a promising future for use in agricultural production.

## 1. Introduction

*Tropaeolum majus* L. (Indian cress) is a traditional, medicinal plant belonging to the Tropaeolaceae family (order Brassicales) [1]. It is considered an extremely valuable plant primarily due to the presence of polyphenols, fatty acids, and the glucosinolate glucotropaeolin and its degradation products (isothiocyanate) [2,3]. Isothiocyanates have become the focus of research in recent years, precisely because of their significant pharmacological, anticancer, antibacterial, antiadhesive and anti-inflammatory effects [2,4,5,6,7].

The antiproliferative and antibacterial activities of *T. majus* can be attributed to benzyl isothiocyanate (BITC), which is the major degradation product of the glucosinolate glucotropaeolin [2,8]. The major degradation product of glucotropaeolin, BITC, is a volatile compound that suppresses the action of chemical carcinogenesis in various preclinical cancer models and is effective against various sensitive and resistant bacteria [7]. Gram-negative bacteria, such as *Salmonella typhi*, *Escherichia coli*, and *Pseudomonas aeruginosa,* and Gram-positive bacteria, including *Staphylococcus aureus* and *Bacillus cereus,* have been associated with food poisoning or food spoilage [9]. Although BITC has excellent biological activity, due to its low water solubility, volatility, and unpleasant odor, its application in the food industry is still limited [10]. The scratch test assay is a standard technique for testing the wound healing potential of different compounds. Although a variety of plant extracts have traditionally been considered to have positive wound healing potential, wound healing assays using plant materials have not been commonly performed [11,12]. Among plant diseases, viral infections are of particular concern because the arsenal of available means to combat these pathogens is very limited. Modern phytotherapy and innovative approaches are promoting the use of essential oils, hydrosols, and other plant extracts to treat various human diseases and also to protect plants from pathogens. Since plants can tolerate high levels of infection in some cases and have understandably evolved strategies to deal with viral pathogens, some of their specialized metabolites could be effective antiviral compounds. In some previous studies, we identified volatile plant constituents as metabolites that, among numerous other functions, protect plants against viral infections [13,14]. Plant isolates are used to prevent a large number of plant diseases caused by phytopathogenic bacteria, fungi, plant parasitic nematodes, and parasitic and non-parasitic weeds [15]. In addition, natural extracts are increasingly becoming the focus of scientific research, as green prevention strategies support the use of natural resources that can replace synthetic remedies and herbicides in crop protection. Therefore, in recent years, modern advanced techniques such as microwave-assisted extraction (MAE) and microwave hydrodiffusion and gravity (MHG) have been used to isolate biologically active compounds. These extraction methods are more environmentally friendly due to their simplicity, lower energy consumption, and shorter processing time compared to conventional extraction methods [16,17]. The microwave extraction technique applies heat to the extracted plant material indirectly via in situ water, while classical techniques apply heat directly [17]. Using MAE/MHG techniques, three types of samples can be obtained: essential oils/extracts and hydrosols (HYs). As for biologically active compounds, the emphasis is mainly placed on their presence in essential oils and extracts [16]. Hydrosols, also called floral and aromatic waters, are complex mixtures that are mostly used in cosmetics and contain trace amounts of essential oils and other compounds that are soluble in water [18,19]. HYs, as by-products of extraction techniques, are often misclassified as wastewater and neglected/discarded, although they contain very valuable bioactive components [20]. Isolated chemical components from the flowers and leaves of *T. majus* are usually identified by colorimetric reactions and gas chromatographic analysis combined with mass spectrometry [21]. Therefore, because of all the above, the novelty of this research is the first detailed investigation of HYs obtained from *T. majus* seeds, to the best of our knowledge.

Consequently, the aim was the characterization and quantification of volatile compounds present in HYs obtained using two modern extraction techniques: MAE and MHG. The main goal of this research was to test the HYs’ biological activities: cytotoxic activity against three cancer cell lines (cervical cancer cell line (HeLa), human colon cancer cell line (HCT116), human osteosarcoma cell line (U2OS)) and a healthy cell line (RPE1); scratch assays (to measure effects on wound healing) using human keratinocyte cells (HaCaT); antibacterial activity against the growth and adhesion to a polystyrene surface of *S. aureus* and *E. coli*; and antiphytoviral activity against TMV.

## 2. Results

### 2.1. Identification of Volatile Components in Hydrosols from T. majus Seeds

The volatile components present in the HYs isolated using MAE and MHG extraction techniques from *T. majus* seeds were identified by GC-MS (Table 1). The stock solution of volatile compounds (VCs) of the HYs using the MAE and MHG techniques were 1.05 mg/mL and 3.95 mg/mL, respectively. The compounds benzaldehyde (9.91%), BITC (62.29%), and benzyl cyanide (BCN; 15.02%) were main components detected in the HY of *T. majus* isolated using the MAE technique. In the HY of *T. majus* obtained using the MHG extraction technique, the main compounds were *α*-thujene (5.25%), BITC (17.89%), and BCN (65.33%). What is interesting is that the two most important/main components present in the HYs of *T. majus* had a reciprocal relationship when comparing the extracts using MAE and MHG extraction techniques.

### 2.2. Cytotoxic Activity

**Figure 1:**
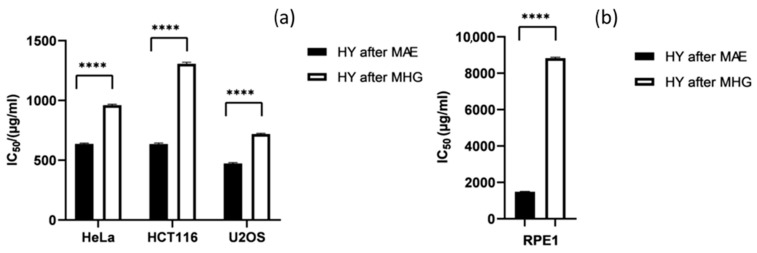
Cytotoxic activity of *T. majus* hydrosols (HYs) isolated using advanced microwave-assisted-extraction techniques, MAE and MHG, on HeLa, HCT116, and U2OS cancer cell lines (**a**) and RPE1 cell line (**b**). Statistical analysis was performed using two-way ANOVA followed by Sidak’s multiple comparisons test. The presented IC_50_ values are mean values of three independent experiments performed in quadruplicate ± SD (standard deviation), and significantly different levels between the two extraction methods are marked as **** *p* < 0.0001.

The cytotoxic activity of the *T. majus* HYs on HeLa, HCT116, and U2OS cancer cell lines was determined for the first time. The results showed that both HYs exhibited moderate activity against the cancer cell lines (Figure 1a). The HY of *T. majus* isolated using MAE (HY after MAE) showed the highest cytotoxic activity with an IC_50_ value of 472.61 µg/mL. The HY obtained using MHG (HY after MHG) had slightly weaker activity on U2OS (IC_50_ = 719.01 μg/mL). The HY isolated using MAE had a similar ability to inhibit the growth of HeLa and HCT116 cells with IC_50_ values of 637.07 μg/mL and 636.13 μg/mL, respectively. In contrast, the HY isolated using MHG were less effective at inhibiting the growth of HeLa and HCT116 cancer cells (IC_50_ = 960.79 μg/mL and IC_50_ = 1307.03 μg/mL, respectively). The healthy cell line (RPE1) showed significant resistance; extremely high concentrations of both HYs were required to inhibit the growth of 50% of the cells (IC_50_ for HY after MAE = 1496.09 μg/mL and IC_50_ for HY after MHG = 8779.88 μg/mL) (Figure 1b).

### 2.3. Scratch Assay

**Figure 2:**
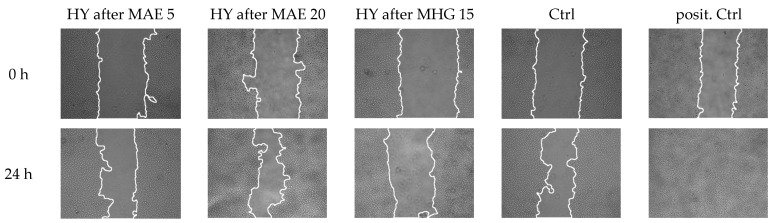
Microphotographs of wound healing assay at timepoints 0 and 24 h after treatment.

The scratch assay (to measure effects on wound healing) was performed by treated HaCaT cells with hydrosols isolated using MAE and MHG extraction techniques at concentrations of 5 µg/mL and 20 µg/mL of the HY isolated using MAE, and 15 µg/mL of the HY isolated using MHG. Both concentrations of the *T. majus* HY isolated using the MAE technique (5 and 20 µg/mL) showed mild and statistically non-significant effects in promoting gap closure compared to untreated cells (Table 2, Figure 2). The *T. majus* HY isolated using MHG at a concentration of 15 µg/mL showed a statistically significant negative effect on wound healing.

### 2.4. Antibacterial Activity

Table 3 presents the results of the antibacterial activity assays of the HYs isolated using MAE and MHG extraction techniques against Gram-positive *S. aureus* and Gram-negative *E. coli*. The stock solutions of the HY isolated using MAE and HY isolated using MHG were at concentrations of 1.05 mg/mL and 3.95 mg/mL, respectively. The obtained results showed that the real MICs for the HY isolated using MAE was above 0.5 mg/mL, and for the HY isolated using MHG, it was above 2 mg/mL (Table 3).

### 2.5. Bacterial Growth Kinetics

To test their effects on bacterial growth, *S. aureus* and *E. coli* were exposed to the HYs isolated from *T. majus* seeds using MAE and MHG extraction techniques for 24 h at concentrations of 0.5 mg/mL for the HYs isolated using MAE and concentrations ranging from 2 mg/mL to 0.5 mg/mL for the HY isolated using MHG (Figure S1). The lack of growth inhibition was visible in the growth curves. The concentration did not significantly affect the growth curve, only slightly lengthening the lag phase.

### 2.6. Antiadhesion Activity

**Figure 3:**
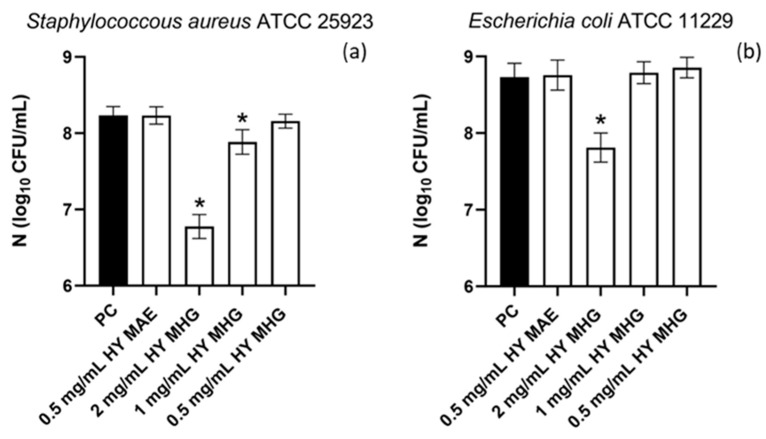
Effects of hydrosols of *T. majus* isolated using MAE and MHG extraction techniques at concentrations of 0.5 mg/mL, 1 mg/mL, and 2 mg/mL on the adhesion of *S. aureus* (**a**) and *E. coli* (**b**) to polystyrene surface. The results are expressed as means ± SD; * *p*-value < 0.05. PC—positive control; HY MAE—hydrosol isolated using MAE extraction; HY MHG—hydrosol isolated using MHG extraction.

The HYs obtained from *T. majus* seeds using MAE and MHG extraction techniques were tested for ability to inhibit the adhesion of *S. aureus* and *E. coli* to a polystyrene surface. The HY isolated using MHG reduced the adhesion of *E. coli* at a concentration of 2 mg/mL, while it also reduced the adhesion of *S. aureus* at a concentration of 1 mg/mL (Figure 3). Due to the stock solution of the HY isolated using MAE (1.05 mg/mL), it was not possible to determine if there was a reduction in adhesion (Figure 3).

### 2.7. Antiphytoviral Activity

**Figure 4:**
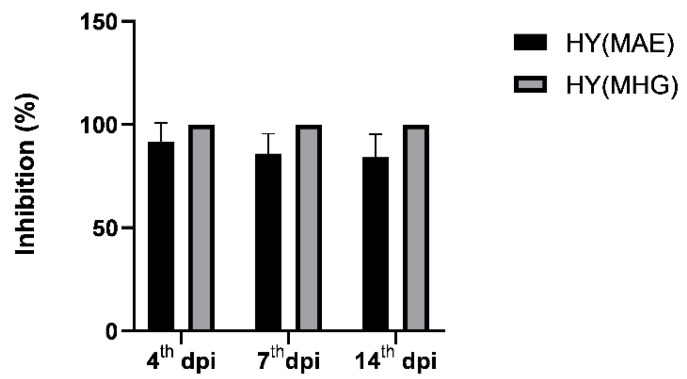
Percentage of inhibition of TMV infection on leaves of treated host plants compared to control plants on the 4th, 7th, and 14th days post inoculation (dpi). Prior to virus inoculation, plants were treated with HYs of *T. majus* for two consecutive days (HY isolated using microwave-assisted extraction (MAE)—HY_MAE_; HY isolated using microwave hydrodiffusion and gravity (MHG) technique—HY_MHG_). The error bars show the standard deviation of the quadruplicate analyses.

With the aim of improving our knowledge of the biological activities of plant volatiles in general, the species *T. majus*, which is still poorly studied in terms of its biological activities, became the focus of our scientific interest. To investigate its antiphytoviral activity, *D. stramonium* plants infected with TMV were pretreated with the HYs of *T. majus* isolated using the MAE and MHG extraction methods. Treatment of the host plants prior to virus inoculation with HYs obtained using both methods significantly inhibited infection, indicating that *T. majus* is a highly effective natural source of antiphytoviral compounds. A comparison of the control and treated plants showed a statistically significant reduction in the number of local lesions (LLN) on HY_MAE_- and HY_MHG_-treated plants. The average LLN on the leaves of the control plants on day 14 after inoculation (dpi) was 12.97, whereas the HY_MAE_-treated plants developed a significantly lower number of lesions during the same period (1.67 lesions per leaf). In addition, HY_MHG_-treated plants in all experimental groups completely reduced the development of lesions, and the leaves of all treated plants showed no symptoms (Table 4). To date, extensive research has been conducted to control TMV, and our results are the first report of complete inhibition of local TMV infection by treatment with *T. majus* HYs. The percent inhibition of virus infection on leaves of HY_MAE_-treated plants at the 4th, 7th, and 14th dpi was 91.65%, 85.89%, and 84.15%, respectively (Figure 4). HY_MHG_-treated plants showed 100% inhibition of virus infection during the same period. Since TMV is one of the most important pathogens of agricultural crops, affecting more than 200 species of herbaceous and, to a lesser extent, woody plants, these results suggest that *T. majus* could be a new and effective source of antiviral compounds in the form of environmentally friendly aqueous solutions.

## 3. Discussion

Many researchers and manufacturers are interested in investigating and including plant extracts in formulations, due to their many and varied biological properties, such as reducing skin pigmentation, skin softening, moisturizing, and promoting wound healing [19]. Therefore, the chemical composition and biological activity of HYs obtained using two extraction methods, MAE and MHG, were studied. Two HYs were obtained and analyzed by GC-MS; the data obtained are shown in Table 1. Also, in this paper, the cytotoxic activity of the HYs against HeLa, HCT116, and U2OS cancer cell lines, and against a healthy cell line (RPE1) was also investigated; the scratch assay (to measure effects on wound healing) was performed using HaCaT cells; the antibacterial activity of HYs was evaluated against the growth and adhesion to a polystyrene surface of *S. aureus* and *E. coli*; and their antiphytoviral activity was determined against TMV. The biological activities investigated in this study focus for the first time exclusively on HYs derived from the seeds of *T. majus.*

The obtained results show that the two main components in HYs, BITC and BCN, have a reciprocal relationship. These two volatiles are breakdown products of the glucosinolate glucotropaeolin [23]. Benyelles et al. reported that glucotropaeolin is the only glucosinolate present in *T. majus*. BITC (82.5%) is the most abundant volatiles compound from the aerial parts (orange flowers, stems, leaves) of *T. majus* from northwest Algeria [24]. The higher BCN content in the HY isolated using MHG can be explained by the presence of an epithiospecific protein due to its interaction with the enzyme myrosinase, which redirects the reaction toward the formation of epithionitrile or nitrile depending on the glucosinolate structure [25]. The epithiospecific protein is heat sensitive and its activity decreases significantly at high temperatures (>50 °C), which is probably the reason for the lower content of BCN and higher content of BITC in the HY isolated using MAE extraction technique [26]. The obtained results agree with those published so far [2,8,27].

Earlier studies by Vrca et al. [2] with an essential oil (EO) and extracts of *T. majus* showed exceptional cytotoxic activity for the EO and slightly lower activity for the extract on cancer cell lines HeLa, U2OS, and HCT116. A chemical analysis revealed that the main volatile components of the EO and extract of *T. majus* are BITC and BCN [2]. BITC showed significantly higher cytotoxic activity on the tested cells compared to BCN. The presence of these compounds is responsible for the biological activity attributed to *T. majus*. Numerous studies have shown that BITC, which is enzymatically hydrolyzed from glucotropaeolin (benzyl glucosinolate), has anti-inflammatory, antioxidant, antiangiogenic, and anticancer effects on various cancers [28,29]. Recently, there has been an increased awareness of the use of natural products and medicines, including treatments for numerous diseases such as cancer, dietary prescriptions, or nutritional supplements. The current treatment options for cancer patients have a number of obstacles, such as high toxicity to normal cells and many other side effects associated with the treatment. On the other hand, biomolecules derived from natural products, such as BITC, offer great potential for cancer treatment [30]. Han et al. demonstrated that BITC can induce apoptosis in gastric adenocarcinoma (AGS) cell lines via a pathway involving ROS-promoted mitochondrial dysfunction and death receptor activation. Kim et al. injected 4T1 breast cancer cells into albino mice (BALB/c) to investigate the effect of oral administration of BITC on tumor growth and metastasis [31]. Significant decreases in tumor growth, hemoglobin content, and vascular endothelial growth factor (VEGF) expression in tumor tissue were observed. BITC also led to a decrease in Bcl-2 protein, an apoptosis inhibitor. Owis et al. prepared an alcoholic extract from the leaves of *T. majus* and tested its potential activity against diethylnitrosamine-induced liver cancer (HCC) in vivo [32]. Oral administration of the extract significantly reduced levels of the inflammatory marker NF-κB and suppressed HCC progression. Oral therapy was combined with 0.5 Gy gamma radiation via the EGF-HER-2 pathway. A histopathological analysis showed a restoration of the liver structure, while an immunohistochemical analysis revealed an increase in pro-apoptotic markers and inhibition of anti-apoptotic factors. The authors proved that *T. majus* can mediate the defense against HCC carcinogenesis in such a way that, in combination with a low dose of gamma radiation, it can stop the further development of the HCC cancer. Phenolic extracts of *T. majus* flowers rich in caffeic acid, coumaric acid, chlorogenic acid, and rutin showed significant cytotoxic activity in synergy with 5-FU (fluorouracil) on the tested MCF -7 breast cancer cell line [33]. Pintão et al. reported the in vitro anticancer properties of BITC against a number of human ovarian cancer cell lines (SKOV-3, 41-M-CHI, CHIcisR), human lung tumor (H-69), murine leukemia L-1210, and a murine plasmacytoma (PC6/sens) [34]. BITC showed significant cytotoxicity at low molar concentrations (0.86 to 9.4 µM) in all the cell lines tested. In the present study, the HYs of *T. majus* isolated using two microwave-assisted extraction methods also predominantly contained glucosinolate degradation products, namely the volatile compounds BITC and BCN. The amount of these compounds in the HYs depended on their water solubility. It is known that BITC and BCN are poorly soluble in water [10], so their concentration in the hydrosol was much lower than in EOs or in the extract, and thus their ability to inhibit the growth of cancer cells as well as healthy cells was lower. Considering the obtained results, the hydrosols of *T. majus* isolated using modern extraction techniques, MAE and MHG, can be considered as a safe natural product that could find application in the cosmetic and food industries.

To our knowledge, there are no previous studies on the wound healing properties of *T. majus* extracts. Only one research study on wound healing using the genus *Tropaeolum* was found. That study was conducted on another species of *Tropaeolum*, namely *Tropaeolum tuberosum*. The authors tested topical preparations containing 1% acidic ethanolic extract of tubers of *T. tuberosum* on mice and demonstrated an improved wound healing activity [35]. Therefore, our research is a pioneer testing of the wound healing potential of *T. majus* HYs. The results of the wound healing test on the HaCaT cell line using different concentrations and extraction methods did not show a positive influence of the *T. majus* HYs; moreover, 15 µg/mL of the *T. majus* HY isolated using MHG showed a negative impact. The HY obtained using MAE extraction at concentrations of 5 µg/mL and 20 µg/mL showed mild and statistically non-significant wound healing effects. These unexpected results may indicate that the method of extraction could be responsible for different effects of HYs on wound healing performance. The concentration of free VCs in both extracts (MAE and MHG) was the main determinant of HY potency. The concentration range used in the wound healing assay of both HYs was similar, and it was determined based on the cytotoxicity results obtained in our study. However, the detailed composition of the MAE and MHG HYs showed several different chemical identities. We believe that the reason for the different healing effects of the HYs should due to the specific composition of each extract. Moreover, the negative impact of the *T. majus* MHG HY on wound healing could be an interesting effect, because other substances that show a negative effect on wound healing, like caffeine [36] and allicin, are considered to be a promising therapeutic candidates for keloid scars [37]. Nevertheless, the reason for the negative effect of 15 µg/mL of the *T. majus* HY isolated using MHG on wound healing remains unclear, and it should be a subject for further studies.

*S. aureus* is the bacterium that is most commonly associated with hospital-acquired wound infections, while *E. coli* is one of the dominant bacteria associated with burn wounds [38]. Due to the increase in bacterial resistance to antibiotics, the focus of research is on biologically active components isolated from plant species used as herbal medicines, due to their ability to produce powerful antibacterial and antifungal compounds [39]. Certain extracts of plant species such as garlic, basil, ginger, sage, mustard, etc., show antimicrobial activity against a wide range of Gram-positive and Gram-negative bacteria [40]. Plants are a rich source of secondary metabolites such as tannins, phenolic compounds, alkaloids, and flavonoids, which have been proven to have antimicrobial properties [41]. Vrca et al. [2] reported antibacterial and antiadhesion activities for EOs and extracts of *T. majus* plants, and for pure BITC and BCN. According to Vrca et al., the EO obtained using MAE and the extract obtained using MHG extraction techniques showed extremely strong antimicrobial activity against *S. aureus* and *E. coli* due to degradation products of gluotropaeolin: BITC and BCN [2]. Consequently, the by-products or HYs obtained from the seeds of *T. majus* were tested for antibacterial activity, bacterial growth kinetics, and antiadhesion activity against *S. aureus* and *E. coli*. The stock solution of the HY isolated using MAE was lower than that isolated using MHG (1.05 mg/mL), and due to the impossibility of testing at higher concentrations, we assume that the MIC concentration is above 0.5 mg/mL. According to the obtained results, the HYs were not effective enough to achieve the true MIC, but the INT color was less intense for 2 mg/mL of the HY isolated using MHG. The HY isolated using MHG at 2 mg/mL reduced the adhesion of *E. coli*, while it also reduced the adhesion of *S. aureus* at 1 mg/mL. Due to the concentration of the stock solution of the HY isolated using MAE (1.05 mg/mL), it was not possible to achieve a reduction in adhesion. The HY isolated using MAE had higher amount of BITC than the HY isolated using the MHG extraction technique, where BCN dominates; according to previous research, ITCs have been proven to be the most biologically active degrading components of glucosinolates [2,42]. Although previous research on HYs has been insignificant in terms of antibacterial activity, according to the research of Nazlić et al., essential oils have a stronger effect compared to HYs, which is consistent with the results of this research [20]. According to Kuete [43], the activity of plant extracts was classified as significant (MIC < 100 µg/ mL), moderate (100 < MIC ≤ 625 µg/mL), or weak (MIC > 625 µg/ mL); thus, *T. majus* HYs have a weak activity. Despite the activity of HYs being weaker than those of EOs when it comes to antimicrobial activity, they definitely have their advantages such as availability, quantity, non-toxicity, and environmentally friendly. Despite the fact that the HYs did not show significant antibacterial activity, they showed excellent antiphytoviral activity.

Plant viruses are important pathogens for agricultural crops, and new antiviral agents are welcome for economic and environmental reasons. Therefore, one of our goals was the investigation of the antiphytoviral activity of *T. majus* HYs, as a continuation of research on the biological activities of this promising and understudied plant species. Our hypothesis of the *T. majus* volatile compounds possessing antiphytoviral activities is supported by the fact that volatiles of various aromatic plant species can stimulate plant defense responses against infections by various pathogens, including viruses [13,14].

Although HYs have emerged in recent years as new potential bioactive candidates to protect plants against viruses [13,44], the use of these environmentally friendly natural products has not been sufficiently explored. In addition to the historical use of HYs in the traditional medicine of Mediterranean countries, they have recently been used in cosmetics and in the food industry to prevent the growth of pathogenic and harmful microorganisms in foods and in the working environment. Glucosinolates (GSLs), water-soluble metabolites found in almost all plants of the order Brassicales, are among the natural volatile chemicals that most likely contribute to plant defenses against pests and diseases. The described antiviral activity of GSLs is mainly focused on their activity against animal viruses [45,46], and a limited number of publications describe GSLs in the context of viral infections of plants [47]. In some of our previous studies and in studies by other authors, plants were treated with HYs to control viral infections, but 100% inhibition of local TMV lesions has not yet been reported. Considering the results already published in the literature [13,48,49], the present results undoubtedly indicate that HYs containing degradation products of the GSL glucotropaeolin as the dominant compound are new and very promising natural sources of antiphytoviral agents.

Although chemical control methods remain essential in the broader context of plant disease control for economic reasons, natural sources should be explored in the development of effective yet environmentally friendly antivirals to reduce the harmful effects of chemicals on the environment. The promising results presented here confirmed our hypothesis about the antiphytoviral activity of the GSLs contained in the HYs of *T. majus*. The reported antiphytoviral activity of both HYs obtained using MAE and MHG extraction techniques deserves more detailed analysis in the future and opens new research areas related to this unexplored bioactivity of GSLs and HYs of *T. majus*.

Based on all the obtained results, in the category of HYs, we can say without a doubt that *T. majus* HYs are one of the most biologically active when compared to other results reported for different plants. Due to their ecological acceptability and the fact that they show biological activity, these HYs have potential applications in the near future in the medicine, pharmaceutical, food, agricultural, and cosmetic industries.

## 4. Materials and Methods

### 4.1. Plant Material and Reagents

The plant materials (*T. majus* L. seeds) were purchased from Marcon d.o.o. (Novi Marof, Croatia). Before the isolation of the volatile compounds present in the HYs, the seeds of *T. majus* were grounded to a fine powder using a coffee grinding machine. Afterward, the milled seeds (ca. 50 g) were soaked in distilled water directly before MAE, and approximately 1 h before MHG extraction. HYs of *T. majus* were obtained using MAE and MHG extraction techniques and an ETHOS X device (Milestone, Milan, Italy) and applying a microwave power of 500 W for a duration of 30 min, as previously described [2,8]. The temperature inside the microwave oven was ca. 98 °C. The HYs were stored at 4 °C until further analysis.

### 4.2. Preparation of the Samples and Analyses of Hydrosols

The *T. majus* HYs (2 samples) obtained using MAE and MHG extraction techniques were prepared for analysis according to Nazlić et al. [50]. Briefly, 2 mL of the HY was added to a glass bottle and capped with a metal cap. The prepared sample was placed in a water bath at a temperature of 40 °C for 20 min due to the fact that the volatile compounds (VCs) evaporate from the water. The process took an additional 20 min to allow the VCs to adsorb to the resin filament of the headspace needle that was injected through the septum of the bottle cap at the beginning of sample preparation. The injection of the HYs was carried out with a headspace injection needle and there was no split ratio (splitless mode). The injection needle collected the volatile compounds from the HY and was then inserted into a GC inlet and left there for 20 min to ensure that all the volatile compounds from the resin filament were resorbed into the injection liner.

### 4.3. Gas Chromatography and Mass Spectrometry

The gas chromatographic analyses of the HY fraction were performed using a gas chromatograph (model 3900; Varian Inc., Lake Forest, CA, USA) equipped with a flame ionization detector and a mass spectrometer (model 2100T; Varian Inc., Lake Forest, CA, USA), capillary column VF-5 ms (30 m × 0.25 mm i.d., coating thickness 0.25 µm, Palo Alto, CA, USA), according to the method described in [50]. The chromatographic conditions were the same as detailed by Dunkić et al. [51]. Briefly, the conditions for the VF-5-ms column were a temperature of 60 °C (isothermal) for 3 min, which was then increased to 246 °C at a rate of 3 °C min^−1^ and maintained for 25 min (isothermal). The conditions for the CP Wax 52 column were a temperature of 70 °C (isothermal) for 5 min, which was then increased to 240 °C at a rate of 3 °C min^−1^ and maintained for 25 min (isothermal). The injection volume was 2 µL and the split ratio was 1:20. The MS conditions were: ion source temperature, 200 °C; ionization voltage, 70 eV; mass scan range, 40–350 mass units. The individual peaks for both HY samples were identified by a comparison of their retention indices of n-alkanes to those of authentic samples and from previous studies [22,52]. The results are expressed as the mean value of three analyses.

### 4.4. Cytotoxic Activity

The cytotoxic activity of the *T. majus* HYs was determined on three cancer cell lines (kindly donated by Prof. Janoš Terzić from the School of Medicine, University of Split, Split, Croatia), HeLa, HCT116, and U2OS, and one healthy cell line, retinal pigmented epithelial cells (RPE1), using the MTS-based CellTiter 96^®^ Aqueous Assay (Promega, Madison, WI, USA) as described in detail by Fredotović et al. [53]. The cells were grown in a CO_2_ incubator at 37 °C and 5% CO_2_ until they reached 80% confluency. The cells were counted using an automated handheld cell counter (Merck, Darmstadt, Germany), seeded into 96-well plates, and treated with serially diluted HYs of *T. majus*. The cells were grown for an additional 48 h, then 20 µL of MTS tetrazolium reagent (Promega, Madison, WI, USA) was added to each well and the plates were left in the incubator at 37 °C and 5% CO_2_ for 3 h. Absorbance was measured at 490 nm using a 96-well plate reader (Infinite M Plex, Tecan, AG, Switzerland). IC_50_ values were calculated from three independent experiments using GraphPad Software Prism 9 (GraphPad, Boston, MA, USA).

### 4.5. Scratch Assay

Human keratinocyte cells (HaCaT) were cultured in Dulbecco’s Modified Eagle’s Medium (DMEM) containing 10% fetal bovine serum (FBS) and 0.1% of an antibiotic–antimycotic solution (penicillin, streptomycin, amphotericin B). HaCaT cells were seeded at a 1 × 10^5^ cells/well density into standard six-well culture plates in complete cell culture medium and maintained at 37 °C, 5% CO_2_ until 80–90% confluency was reached. Prior to the experiment, the cells were starved for 24 h in serum-free media (to inhibit cell proliferation). For the scratch assay, a sterile 200 μL pipette tip was used to make a straight scratch in the monolayer of cells, simulating an epithelial wound. After the scratch was made, the cellular debris was washed out with Dulbecco′s phosphate-buffered saline (DPBS). Growth medium containing different concentrations and of the two *T. majus* HYs (5 µg/mL HY isolated using MAE, 15 µg/mL HY isolated using MGH and 20 µg/mL HY isolated using MAE) was added to each well, followed by incubation for 24 h. Negative control wells were kept in HY-free growth media. Positive control wells were kept in complete growth media containing 5% FBS. An inverted microscope (Olympus IX73, Olympus, Tokyo, Japan) equipped with a digital camera was used to obtain images of the wound healing assay. Two representative images from different parts of the scratched area for each replicate well were digitally photographed. Wound closure was monitored at time 0 h and 24 h after the scratch. ImageJ software 1.8.0. (NIH, Bethesda, Rockville, MD, USA) was used to measure the size of the wound area. The closure of the wound area was measured and expressed as a percentage of the initial wound area (determined at 0 h) using the following formula:

S _cell free area_ = [(Area_t0_−Area_t24_)/Area_t0_] × 100,

For the statistical analysis, the Mann–Whitney U test was used (Statistica 14.1.0, Tibcosoftware Inc., Palo Alto, CA, USA). Significance was defined as *p* < 0.05.

### 4.6. Antibacterial Activity

Gram-positive *S. aureus* ATCC 25,923 and Gram-negative *E. coli* ATCC 11,229 were used in this study. The bacterial strains were stored at −80 °C in tryptic soy broth (Biolife, Milan, Italy) together with 15% glycerol (Kemika, Zagreb, Croatia), revitalized on Mueller–Hinton (MH) agar (BioMéroeux, Marcy-l’Étoile, France), and incubated at 37 °C in aerobic conditions for 24 h for the antibacterial activity assay.

### 4.7. Antibacterial Susceptibility

To determine the minimal inhibitory concentrations (MICs), the microdilution method was used according to Klančnik et al. and the EUCAST guideline [54,55]. Briefly, the HYs were dissolved and filtered through 0.22 µm filters (Sartorius, Croatia) to ensure that the HYs were sterile. Two-fold serial dilutions of the HYs were performed in a 96-well microtiter plate to achieve concentrations from 2 mg/mL to 0.5 mg/mL in a final volume of 50 µL. A 50 µL volume of prepared inoculum (10^5^ CFU/mL) was added to each well and mixed. A 10 µL volume of 2-*p*-iodophenyl-3-*p*-nitrophenyl-5-tetrazolium chloride (INT, Sigma Aldrich, St. Louis, MO, USA) was added after incubation and was used as an indicator for bacterial metabolic activity [54].

### 4.8. Bacterial Growth Kinetics

The HYs were added to 5 mL of growth medium to give final concentrations of 2 mg/mL to 0.5 mg/mL for *S. aureus* ATCC 25,923 and *E. coli* ATCC 11,229. *S. aureus* or *E. coli* cultures without the addition of *T. majus* HYs were used as a positive control. As a negative control, MH broth with or without the addition of the *T. majus* HYs at different concentrations was used and was deducted from the results obtained from the experimental samples. The inocula were prepared as described above. A total of 100 µL of the prepared cultures and negative controls, with or without the addition of HYs, were added to 96-well microtiter plates (Nunc 266 120 polystyrene plates; Nunc, Denmark). The absorbance was measured at 600 nm using a Multiskan reader (Thermo Scientific, Waltham, MA, USA) every 30 min over 24 h at 37 °C to obtain growth curves.

### 4.9. Antiadhesion Assay

The adhesion of *S. aureus* ATCC 25,923 and *E. coli* ATCC 11,229 was analyzed with after treatment with the *T. majus* HYs obtained using MAE and MHG extraction techniques. The inocula were prepared as described above and treated with HYs at MIC, ½ MIC, and ¼ MIC concentrations. The treated inocula (200 µL) were then transferred to 96-well polystyrene microtiter plates (Nunc 266 120 polystyrene plates; Nunc, Denmark) and incubated at 37 °C under aerobic conditions for 24 h. To remove non-adherent cells, each well in the microtiter plate was rinsed three times with phosphate-buffered saline (PBS) (Oxoid, Hampshire, UK); afterward, 200 µL of PBS was added to each well and the plates were sonicated for 10 min (28 kHz, 300 W; IskraPIo, Šentjernej, Slovenia). CFU/mL was used to measure the adhesion of the cells, as previously described by Šikić Pogačar et al. [56]. The negative control was an untreated culture. The experiments were carried out in triplicate as three or more independent experiments. The data are presented as means ± standard deviation (SD); the analysis was performed using GraphPad Software Prism 9 (Boston, MA, USA). IBM SPSS Statistics 23 (Statsoft Inc., Tulsa, OK, USA) was used to the perform statistical analyses. The Kolmogorov–Smirnov test of normality was used to determine the distribution of the data and statistical significance was determined using *T*-tests for two independent means. Data with a *p*-value < 0.05 were considered significant.

### 4.10. Antiphytoviral Activity Assay

The antiphytoviral activity of *T. majus* HYs (HY isolated using MAE—HY_MAE_; HY isolated using the MHG techniques—HY_MHG_) was tested using a local host plant, the species *Datura stramonium* L., which was infected with tobacco mosaic virus (TMV). An inoculum prepared from systemically infected leaves of *Nicotiana tabacum* L. cv. Samsun was diluted with phosphate buffer to obtain 10 to 20 lesions per inoculated leaf of the local host plant. HY (undiluted) was applied as a spray solution to the leaves of *D. stramonium* on two consecutive days before virus inoculation. Antiphytoviral activity was evaluated as the percentage inhibition of the number of local lesions on the leaves of treated and control plants [52]. Statistical analysis was performed using GraphPad Prism version 9. All data are presented as mean ± SD (*n* = 4). Statistical significance was determined by multiple *t*-tests [14].

## 5. Conclusions

HYs are environmentally friendly, non-toxic by-products of advanced and conventional extraction techniques that are often unjustly neglected despite possessing biologically active constituents. The HYs of *T. majus* obtained using MAE and MHG extraction techniques are enriched with volatile components: *α*-thujene, benzaldehyde, benzyl isothiocyanate (BITC), and benzyl cyanide (BCN). The HY obtained using MAE showed better cytotoxic activity against three cancer cell lines (HeLa, HCT116, and U2OS) than the HY isolated using MHG. The healthy cell line, retinal pigmented epithelial cells (RPE1), showed extremely high resistance to both HYs. The MICs of the *T. majus* HY isolated using MAE was above 0.5 mg/mL, and for the *T. majus* HY isolated using MHG, it was above 2 mg/mL. The HY isolated using MHG reduced the adhesion of *E. coli* at a concentration of 2 mg/mL, while it also reduced the adhesion of *S. aureus* at a concentration of 1 mg/mL. Due to the stock solution of the HY isolated using MAE, it was not possible to determine the concentration at which it reduces adhesion. Moreover, both HYs showed high antiphytoviral activity against TMV, achieving 100% inhibition of local lesions, which is the first such result of inhibition of TMV viral infection by an HY. Due to the significant and important results on the antiphytoviral activity, HYs of *T. majus* have a potential future in agricultural production.

The advantages of HYs, in addition to showing a wide spectrum of biological activity, is their ecological acceptability due to their non-toxicity, lack of organic solvents in the extraction process, and a larger volume compared to essential oils and extracts, which enables them to be more desirable for use in the medicine, pharmaceutical, food, agriculture, and cosmetic industries. In the category of HYs, we can say without a doubt that *T. majus* HYs are one of the most biologically active.

## Figures and Tables

**Table 1:** Chemical composition of volatile compounds in hydrosols of *T. majus* isolated using microwave-assisted extraction (MAE) and microwave hydrodiffusion and gravity (MHG).

Volatile Compound in Hydrosols of *T. majus*	RI	MAE (%)	MHG (%)
*α*-Thujene	924	1.71	5.25
Benzaldehyde	952	9.91	1.86
Benzene acetaldehyde	1036	0.22	0.43
Benzyl cyanide (BCN)	1140	15.02	65.33
Carvone	1241	0.25	2.31
Benzyl isothiocyanate (BITC)	1371	62.29	17.89
Methyl eugenol *	1403	1.59	1.11
Caryophyllene oxide *	1581	2.51	–
Total identification (%)		93.5	94.18

Retention indices (RIs) were determined relative to a series of n-alkanes (C8–C40) on capillary columns VF5-ms (RI). Identification method: RI, comparison of RIs with those in a self-generated library reported in the literature [22] and/or with authentic samples; comparison of mass spectra with those in the NIST02 and Wiley 9 mass spectral libraries. * injection of reference compounds; –, not identified.

**Table 2:** Percentage of cell-free area 24 h after treatment with *T. majus* hydrosols isolated using microwave-assisted isolation (MAE) and microwave hydrodiffusion and gravity (MHG). *p* value was calculated in comparison with untreated cells (Ctrl) with * *p* < 0.05 considered significance.

*T. majus* L. HY	Concentration (µg/mL)	% Cell-Free Area	SEM	*p* Value
HY isolated using MAE	5	63.40	4.69	*p* > 0.05
HY isolated using MAE	20	61.41	6.53	*p* > 0.05
HY isolated using MHG	15	77.10	3.02	* *p* < 0.05

**Table 3:** Minimal inhibitory concentration (mg/mL) against *S. aureus* ATCC 25,923 and *E. coli* ATCC 11,229 for hydrosols of *T. majus* isolated using microwave-assisted isolation (MAE) and microwave hydrodiffusion and gravity (MHG).

*T. majus* Samples	*S. aureus* ATCC 25,923	*E. coli* ATCC 11,229
	γ/(mg/mL) MIC	γ/(mg/mL) MIC
HY isolated using MAE	>0.5	>0.5
HY isolated using MHG	>2	>2

**Table 4:** Number of local lesions on leaves of local host plants treated with hydrosols (HY_MAE_ and HY_MHG_) of *T. majus* before virus inoculation.

dpi	LLN ± SD		
	C	PT-HY_MAE_	PT-HY_MHG_
4th	10.63 ± 0.98	0.89 ± 1.08 ***	0 ± 0.0 ***
7th	9.83 ± 1.24	1.39 ± 1.16 ***	0 ± 0.0 ***
14th	10.51 ± 1.27	1.67 ± 0.92 ***	0 ± 0.0 ***

LLN, number of local lesions; dpi, day post inoculation; C, leaves of control plants; PT-HY_MAE_, leaves of plants pretreated with hydrosol isolated using the microwave-assisted extraction; PT-HY_MHG_, leaves of plants pretreated with hydrosol isolated using microwave hydrodiffusion and gravity technique; SD, standard deviation of quadruplicate analyses. Significant differences were determined by multiple *t*-tests; *** statistically significant differences between control and treatment data (*p* ˂ 0.0001).

## Data Availability

The samples and any additional research data are available from the authors on request.

## Supplementary Materials

The following supporting information can be downloaded at: https://www.mdpi.com/article/10.3390/plants12223897/s1, Figure S1: Effects of hydrosols of *T. majus* in different concentrations (mg/mL) on the growth of *S. aureus* (**a**) and *E. coli* (**b**). Cultures were aerobically incubated for 24 h at 37 °C. Negative controls were deducted from the obtained results. Average values of A600 nm ± SD are shown.
