# Supplementary material for: Significant Benefits of Environmentally Friendly Hydrosols from Tropaeolum majus L. Seeds with Multiple Biological Activities
Source: Plants (Basel). 2023 Nov 18;12(22):3897. doi: 10.3390/plants12223897 (PMC10675760; doi:10.3390/plants12223897)
Supplement: Supplementary file 1 [file plants-12-03897-s001.zip › plants-2665754-supplementary.pdf]

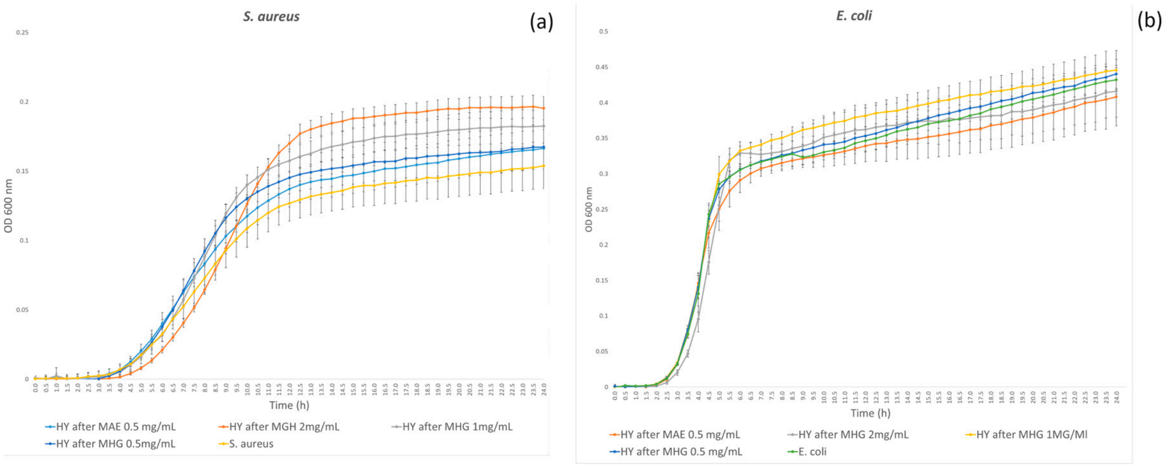

Figure S1. Effects of hydrosols of *T. majus* in different concentrations (mg/mL) on the growth of *S. aureus* (**a**) and *E. coli* (**b**). Cultures were aerobically incubated for 24 h at 37 °C. Negative controls were deducted from the obtained results. Average values of A600 nm ± SD are shown.
